# A national survey on routines regarding sedation in Swedish intensive care units 

**DOI:** 10.1080/03009734.2019.1616339

**Published:** 2019-05-23

**Authors:** Oskar Talsi, Ritva Kiiski Berggren, Göran Johansson, Ola Winsö

**Affiliations:** a Department of Surgical and Perioperative Sciences, Anaesthesiology and Intensive Care Medicine, Umeå University, Umeå, Sweden;; b Swedish National Quality Registry for Intensive Care (SIR), Karlstad, Sweden

**Keywords:** Analgesics, intensive care, mechanical ventilation, sedation scale, sedatives

## Abstract

**Background:** Previous studies concerning sedation in Swedish intensive care units (ICU) have shown variability in drug choices and strategies. Currently, there are no national guidelines on this topic. As an update to a Nordic survey from 2004, and as a follow-up to a recently introduced quality indicator from the Swedish Intensive Care Registry, we performed a national survey.

**Methods:** A digital survey was sent to the ICUs in Sweden, asking for sedation routines regarding hypnosedatives, analgosedatives, protocols, sedation scales, etc.

**Results:** Fifty out of 80 ICUs responded to the survey. All units used sedation scales, and 88% used the RASS scale; 80% used written guidelines for sedation. Propofol and dexmedetomidine were the preferred short-term hypnosedatives. Propofol, dexmedetomidine, and midazolam were preferred for long-term hypnosedation. Remifentanil, morphine, and fentanyl were the most frequently used agents for analgosedation.

**Conclusions:** All ICUs used a sedation scale, an increase compared with previous studies. Concerning the choice of hypno- and analgosedatives, the use of dexmedetomidine, clonidine, and remifentanil has increased, and the use of benzodiazepines has decreased since the Nordic survey in 2004.

## Introduction

Sedation is crucial for treatment in the intensive care unit (ICU). The concept is wide and includes everything from light sedation with patients being awake and able to communicate, to heavy sedation where patients cannot manage their own respiration. The purpose of sedation from the patient perspective is relief of pain and anxiety, which means that nursing care, communication, and drugs interact to achieve these goals. Nowadays, the general opinion on sedation among ICU staffs and recent studies is pointing towards less sedation. Today more focus is directed on analgesia, since heavy sedation has been reported to prolong the length of stay (LOS) at the ICU and to increase the rate of complications (1–5). Lower levels of sedation also permit easier evaluation of disease progress, easier follow-up of analgesic therapy, and they simplify clinical examinations.

Previous studies have shown that the level of sedation in the ICU has a tendency to be too deep and that the analgesic therapy is insufficient (6). Nowadays it is well known that monitoring of sedation levels and utilization of validated sedation scales, with or without a sedation protocol, give a more accurate sedation level. More accurate and lighter sedation gives shorter LOS, shorter time on mechanical ventilation, and fewer complications in the ICU (7,8). Too heavy sedation can also contribute to an increase of post-traumatic stress disorder (PTSD) symptoms in ICU survivors, though many aspects are involved (9). Earlier Swedish and Nordic observational studies from 2003 and 2004 have shown varying results in usage of sedation scales and goals, ranging from 16% to about 50% (10,11). Similar studies in Europe and North America show data comparable to the use of sedation scales in the Nordic countries (12–14).

Subjective scales evaluating the level of sedation are considered more accurate than objective measures of brain activity, e.g. bispectral index (BIS) (1). There are multiple sedation scales in clinical use, though a ‘gold standard’ has not been identified. The recommendations today promote the use of a validated scale, e.g. the Richmond Agitation Sedation Scale, which is one of the most frequently used sedation scales in Europe (1,15–18).

ICU care is expensive, and the number of beds is often very limited. It is therefore relevant, both for patients and hospitals, that knowledge on strategies which can reduce LOS and complications is used. Utilization of sedation scales and definition of sedation goals are some of the Swedish Intensive Care Registry’s eight quality indicators, but there are currently no national guidelines on which sedation scale to use. Moreover, there are no recent Swedish studies on the compliance to this new quality indicator.

Since there are no written national guidelines on this subject in Sweden, the type of treatment varies between hospitals. Previous studies have shown that propofol and midazolam were the most frequently used hypnosedatives in Sweden (10). Recently, the use of dexmedetomidine was promoted due to the shorter time on mechanical ventilation and shorter LOS at the ICU (19).

The aim of this study was to investigate the current sedation practice in Sweden based on a national survey. Focus in the survey was on the use of sedation scales, drug administration methods, and choice of pharmacological agents for sedation and analgesia.

## Material and methods

A national survey was created in cooperation with the Swedish National Quality Registry for Intensive Care (SIR). The survey was sent out on 12 April 2017 to 80 ICUs in Sweden, through SIR’s member register. The survey was an electronic questionnaire consisting of multiple-choice questions, with a possibility to add written answers at the end. In the survey, we asked for routines regarding the use of sedation scales, sedation goals, and preferred analgosedatives and hypnosedatives (see Appendix). The respondent was asked to rank the three most frequently used hypnosedatives and analgosedatives. Additional data were assembled through email.

### Ethics

It was voluntary to participate in the study, and a written reply was considered as informed consent. The principal study concept was approved by the local ethical committee on 10 April 2018 (EPN 2018/13-31).

### Statistical analysis

IBM SPSS Statistics (version 24.0) was used. The Mann–Whitney test and the Kruskall–Wallis test were used for statistical calculations; 95% confidence interval was used, and significance level was set at *P* < 0.05.

## Results

The total number of responders was 50 out of 80 (63%) surveys sent out. The responding units were categorized according to the Swedish Intensive Care Society (SIS) classification from 1 to 3, where 3 is the most advanced level of ICU. Eight of the responders were from category 1; 29 from category 2; and 13 from category 3. Median number of ventilators possible to use simultaneously per ICU was 8 (min. 2, max. 18).

All the responding units used a sedation scale. Forty-four (88%) of the units used the RASS scale, making it the predominantly used sedation scale. The Motor Activity Assessment Scale (MAAS) was used by four units; one paediatric ICU used the Comfort B scale; and one unit used an unspecified scale. Local written guidelines were used by 80% of the responding ICUs, and 10% were working on such guidelines. Daily ‘wake-up calls’ were used in 58% of the units.

The two most frequently used hypnosedatives for short-term sedation (<24 h) were propofol (78%) and dexmedetomidine (20%). The most popular combination was to use propofol as first choice and dexmedetomidine as second choice. For long-term hypnosedation (>24 h) propofol (52%) was the most frequently used agent. The most popular combination was to use propofol as first choice and dexmedetomidine as second choice. There is one more first choice than responders in the category long-term sedation, because one respondent gave two drugs as first choice (Table 1). For analgosedation remifentanil (36%), morphine (30%), and fentanyl (26%) were the preferred agents. The most popular combination of analgosedatives was to use remifentanil or morphine as first choice and ketobemidon or fentanyl as second choice (Table 2). In one part of the survey we asked for the use of alpha-2-agonists. All the responding units use dexmedetomidine. The most common indications for dexmedetomidine were non-invasive ventilation (88%), shallow sedation (88%), and nightly sedation (80%). The most common indications for clonidine were shallow sedation (RASS > -2) (64%), non-invasive ventilation (46%), nightly sedation, and deep sedation (RASS < -2) (32%) (Table 3).

**Table 1.** Hypnosedation, short- and long-term use.

Choice	First	Second	Third
Short term use, <24 hours			
Propofol	39	10	0
Dexmedetomidine	10	31	6
Clonidine	1	5	10
Diazepam	0	1	0
Esketamine	0	0	6
Lorazepam	0	0	0
Midazolam	0	1	18
Other	0	2	4
Long term use, >24 hours			
Propofol	26	15	6
Dexmedetomidine	16	16	5
Midazolam	7	8	12
Clonidine	2	7	14
Diazepam	0	0	0
Esketamine	0	1	2
Lorazepam	0	1	2
Thiopental	0	0	2
Other	0	0	2

Data presented as preferred choices from the participating national Swedish ICUs.

**Table 2.** Analgosedation.

Choice	First	Second	Third
Remifentanil	18	6	9
Morphine	15	6	11
Fentanyl	13	12	3
Ketobemidone	3	9	5
Alfentanil	0	4	6
Esketamine	0	1	5
Clonidine	0	6	5
Sufentanil	0	0	0
Other	1	4	1

Data presented as preferred choices from the participating national Swedish ICUs.

**Table 3.** Indications for the use of dexmedetomidine and clonidine.

	*n*
Dexmedetomidine	
Non-invasive ventilation	44
Sedation (RASS > -2)	44
Nightly sedation	40
Sedation (RASS < –1)	7
Short procedures	6
Other	5
Clonidine	
Sedation (RASS > –2)	32
Non-invasive ventilation	23
Nightly sedation	18
Sedation (RASS < –1)	16
Abstinence	9
Weaning	6
Short procedures	5
Do not use	4
Hypertension	3
Other	2

Data presented as preferred choices from the participating national Swedish ICUs.

Continuous infusion was the preferred administration method for all the responding units (50/50) in the category hypnosedation. Thirty-one out of 50 used intermittent intravenous injections as second choice. In the category analgosedation, 45 out of 50 (90%) used continuous infusions as first choice, and four out of 50 units (8%) used intermittent injections as first choice. The most frequently used method for follow-up on sedation level was that nurses and assistant nurses examined the patient (40/50, 80%). In this group of 40 answers, 31 (78%) made a note in the patient chart and reported at rounds; five units only made a note in the patient chart; and four units (10%) only reported at rounds.

## Discussion

Of the responding units 80% used written guidelines for sedation, an increase from 44% in 2004 (10). All the responding ICUs used a sedation scale, which also is an increase from 2004 when 54% of Swedish ICUs responded that they used a sedation scale; 100% is a high number and might not fully represent the situation nationwide, since there were 30 units missing in the survey. But comparable numbers have been observed in a recent similar study from Great Britain (20). Eighty-eight per cent of the ICUs used the RASS scale, making it the predominantly used sedation scale. This is a change from the 2004 survey, where MAAS was the most frequently used scale (58%) and no use of the RASS scale was registered. Most probably, the increase in usage of sedation scales reflects the current state of knowledge on sedation in ICU settings. Furthermore, the increase in utilization of the RASS scale might be due to ease of use and that it perhaps is more detailed in the span of light sedation. The RASS scale is recommended in both American and German guidelines and was the most frequently used sedation scale in a recent British ICU survey (1,18,20).

In our survey, it was possible to identify two favourite drugs in the category hypnosedation, propofol and dexmedetomidine. These drugs were preferred in both the category short- and long-term sedation. However, in the category long-term sedation, midazolam was preferred as first choice by 14% of the responders. The change of use in both short- and long-term hypnosedation reflects recent studies, promoting a lighter level of sedation and supporting a non-benzodiazepine sedation strategy (18,19). This pattern has also been observed in recent studies and guidelines, where deep sedation is recommended only in specific patient groups (1,18).

It was difficult to identify a clear favourite in the category analgosedation, but remifentanil was preferred by most responders. This is a change from 2004 when no use of remifentanil was registered (10), partially because it was a relatively new drug at that time. Due to its pharmacokinetic properties, remifentanil is mostly administered as an infusion. Because of its short-acting nature, it needs to be withdrawn carefully. The short-acting nature makes it practical for patients with renal or hepatic failure, which is often the case in the ICU (21). Remifentanil is also well tolerated by ICU patients, facilitates a fast and predictable extubation, and may shorten ICU LOS and time on mechanical ventilation (22).

Continuous infusions were preferred by all ICUs in the category hypnosedation and by most responders in the category analgosedation, even though continuous infusions have certain drawbacks. Thus, continuous infusions of benzodiazepines have been associated with higher risk of delirium (1,23). Continuous intravenous sedation has also been linked to prolonged ICU LOS and increased duration of mechanical ventilation and hospitalization (24). One explanation to the high reported use of continuous infusions might be the increased use of remifentanil and dexmedetomidine, drugs only administered as intravenous infusions.

The responses in the survey reflect the current state in Swedish ICUs. The response rate (63%) is comparable to similar studies elsewhere in Europe (10,13,17,25). However, this survey does not say anything about quantities of the drugs used and actual compliance to sedation scales and local guidelines. Our survey was filled out by one physician for each ICU, and there might be a discrepancy from the actual clinical practice. A similar survey carried out in 2013 (25), sent out to ICU nurses in Europe, recorded no answers with dexmedetomidine as the preferred first choice sedative. This raises questions on whether there has been a shift in drug choice over a short period of time or if the answers in this or previous studies over- or underestimate the use of dexmedetomidine. To get a more representative picture of the current practice in Swedish ICUs, prospective multicentre studies or point prevalence studies on site need to be performed.

To conclude, we observed a few changes from the study published in 2004 (10). All the responding units use sedation scales, and the preferred scale is RASS. Similar changes are reported from other countries, e.g. the UK (17,20), and are supported by international guidelines (1,18). Dexmedetomidine and clonidine are used regularly. Remifentanil was reported as the first-choice analgesic agent, even though a clear favourite in this category was not possible to identify.

## Appendix

The questionnaire sent to the ICUs was designed as a multiple-choice form with the possibility to add written answers. Background data were also asked for.

*Questions:*

1. Do you use written guidelines for sedation?
2. Which sedation scale do you use?
3. Do you use daily interruption of sedation, so-called ‘Wake up-calls’?
4. How are drugs for hypnosedation administered?
5. How are drugs for analgosedation administered?
6. Which are the main drugs used for short-term hypnosedation (<24 h)?
7. Which are the main drugs used for long-term hypnosedation (>24 h)?
8. Which are the main drugs used for analgosedation?
9. To which patient groups/situations do you use dexmedetomidine?
10. To which patient groups/situations do you use clonidine?
11. Are you reporting data to SIR, according to SIR’s quality indicator for sedation scales and sedation goals?
